# Supplementary material for: Accurate identification of bacteriophages from metagenomic data using Transformer
Source: Brief Bioinform. 2022 Jun 30;23(4):bbac258. doi: 10.1093/bib/bbac258 (PMC9294416; doi:10.1093/bib/bbac258)
Supplement: Supplementary_file_1_bbac258 [file supplementary_file_1_bbac258.pdf]

Visualization of the Size distribution of the protein clusters

The sizes of different clusters can be quite different as shown in FigS. 1. Due to the similarity cutoff, proteins of similar functions may be divided into multiple clusters.

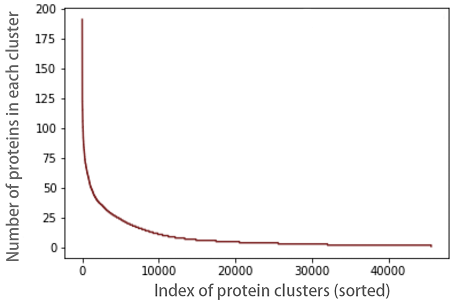

FigS. 1: Size distribution of the protein clusters. X-axis: protein clusters sorted by the number of proteins in the cluster. Y-axis: the number of proteins in each cluster.

Visualization of the attention score

To examine whether the self-attention mechanism learns the protein associations, we visualized the attention score matrix ($QK^T \in \mathbb{R}^{len \times len}$) following the method introduced in [5, 6]. First, we used the overall min-max normalization on the attention score to scale the range to [0, 1]. Then, we visualized the attention score using a heat map.

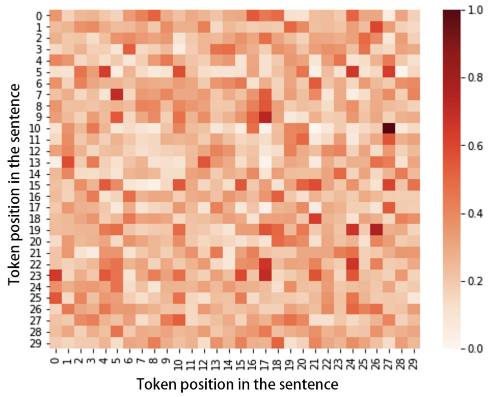

FigS. 2: Heat map of the attention score matrix. The higher the value, the stronger the associations. X-axis and Y-axis: The first 30 token positions in the sentence.

A matrix for an input phage contig is visualized using a heat map in FigS. 2. According to the definition of the attention score, bigger values (darker colors) indicate stronger association between the corresponding tokens (protein clusters) indexed at the row and column. This heat map only contains the attention scores for the first 30 proteins for better visualization. The padded zeros are not shown in this figure. The position of the highest value in FigS. 2 is (27, 10). Although the matrix is not symmetric, both (10, 27) and (27, 10) have the highest value in the 10th and 27th row, respectively, suggesting that the 27th token has a strong association with the 10th token. Our protein vocabulary shows that these two tokens are protein cluster PC_002077 and PC_000567, which contain proteins gp54 and gpE. According to the protein function annotation by Uniprot [1], gp54 is the baseplate protein that forms the baseplate-tail tube junction, and gpE is the tail assembly protein. These two proteins together play essential roles in the phage structure construction. Altogether, there are 39 and 64 proteins in PC_002077 and PC_000567, respectively. 33 phage genomes in our test set contain both PC_002077 and PC_000567. The attention scores between PC_002077 and PC_000567 for the 33 phage genomes are all higher than the average value and four of them have the highest scores. This experiment suggests that the self-attention mechanism can learn important protein associations with biological significance.

Experiments on the simulated data

In this experiment, we use CAMISIM [2] to simulate a small scale metagenomic dataset. We choose commonly seen bacteria living in the human gut and the phages that infect these bacteria. CAMISIM, which can flexibly model community structure and sequencing data properties, can generate simulated data functionally close to the real data. We used CAMISIM to generate 2×270bp paired-end Illumina reads based on log-normal distribution abundance profiles. We simulated the phages' and hosts' reads separately and then combined them to control their proportion in the data. We generated three datasets with the phage/host ratio being 7: 3, 5:5, and 3:7, respectively. The size of each data is 1 GB. The name of the bacteria and the number of phages that infect the bacteria are listed in Table. 1.

**Bacteria species**	Number of phages
Butyrivibrio fibrisolvens	1
Bacteroides fragilis	2
Bacteroides xylanisolvens	1
Butyrivibrio fibrisolvens	6
Faecalibacterium prausnitzii	8
Parabacteroides merdae	1
Escherichia coli	172

Table 1: Information of the bacterial genomes and the infecting phages.

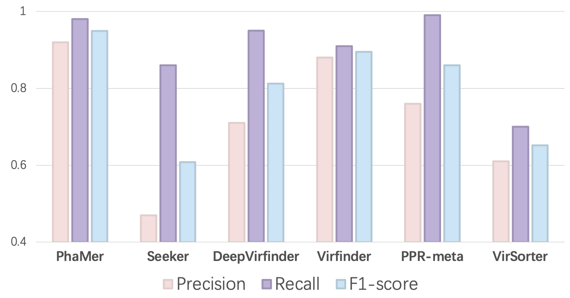

FigS. 3: Phage detection performance on the simulated data generated by CAMISIM. X-axis: the names of the tested methods. Y-axis: the scores of three metrics (precision, reacall, and F1-score). The reported performance is averaged on the three sets with phage vs. bacteria ratio of 7:3, 5:5, 3:7.

After simulating the reads from the reference genomes, we used metaSPAdes [4] to assemble the reads into contigs and removed contigs < 3kbp. To assign labels to the contigs, we used MetaQUAST [3] to map contigs to reference genomes and calculated the coverage. Only those contigs with at least 80% of the sequence aligning to a reference genome are kept. Others are likely chimeric contigs due to assembly errors and thus are not used for testing. Then we run all five tools to identify phage contigs from this simulated data. The average performance of the three samples is shown in FigS. 3. The comparison reveals that our method achieved the best precision and F1-score on the simulated dataset. Compared to the benchmark dataset, the precision of all other tools decreases on these assembled contigs. PhaMer still maintains high precision. PPR-meta's recall is 0.99, which is 1% higher than PhaMer. But its precision is only 0.76, 16% lower than PhaMer.

References

1. UniProt Consortium. UniProt: a worldwide hub of protein knowledge. *Nucleic Acids Research*, 47(D1):D506–D515, 2019.
2. Adrian Fritz, Peter Hofmann, Stephan Majda, Eik Dahms, Johannes Dröge, Jessika Fiedler, Till R Lesker, Peter Belmann, Matthew Z DeMaere, Aaron E Darling, et al. CAMISIM: simulating metagenomes and microbial communities. *Microbiome*, 7(1):1–12, 2019.
3. Alla Mikheenko, Vladislav Saveliev, and Alexey Gurevich. MetaQUAST: evaluation of metagenome assemblies. *Bioinformatics*, 32(7):1088–1090, 2016.
4. Sergey Nurk, Dmitry Meleshko, Anton Korobeynikov, and Pavel A Pevzner. metaSPAdes: a new versatile metagenomic assembler. *Genome Research*, 27(5):824–834, 2017.
5. Tim Rocktäschel, Edward Grefenstette, Karl Moritz Hermann, Tomáš Kočiskỳ, and Phil Blunsom. Reasoning about entailment with neural attention. In *4th International Conference on Learning Representations(ICLR)*. Engineering and Technology organization, 2016.
6. Jesse Vig. A multiscale visualization of attention in the transformer model. *arXiv preprint arXiv:1906.05714*, 2019.
